# Peptide Inhibitors of Vascular Endothelial Growth Factor A: Current Situation and Perspectives

**DOI:** 10.3390/pharmaceutics13091337

**Published:** 2021-08-26

**Authors:** Ivan Guryanov, Tatiana Tennikova, Arto Urtti

**Affiliations:** 1 Institute of Chemistry, St. Petersburg State University, Universitetsky pr. 26, Peterhof, 198504 St. Petersburg, Russia; 2 Division of Pharmaceutical Biosciences, University of Helsinki, Viikinkaari 5 E, 00014 Helsinki, Finland; arto.urtti@helsinki.fi; 3 School of Pharmacy, University of Eastern Finland, Yliopistonranta 1 C, 70211 Kuopio, Finland

**Keywords:** VEGF, VEGFA, VEGFR, peptide, affinity, binding, angiogenesis

## Abstract

Vascular endothelial growth factors (VEGFs) are the family of extracellular signaling proteins involved in the processes of angiogenesis. VEGFA overexpression and altered regulation of VEGFA signaling pathways lead to pathological angiogenesis, which contributes to the progression of various diseases, such as age-related macular degeneration and cancer. Monoclonal antibodies and decoy receptors have been extensively used in the anti-angiogenic therapies for the neutralization of VEGFA. However, multiple side effects, solubility and aggregation issues, and the involvement of compensatory VEGFA-independent pro-angiogenic mechanisms limit the use of the existing VEGFA inhibitors. Short chemically synthesized VEGFA binding peptides are a promising alternative to these full-length proteins. In this review, we summarize anti-VEGFA peptides identified so far and discuss the molecular basis of their inhibitory activity to highlight their pharmacological potential as anti-angiogenic drugs.

## 1. Introduction

The enhancement of the efficiency, selectivity, and targeting of the pharmaceuticals to the desired biomolecules and cells is one of the main objectives of modern drug design. Despite progress in the understanding of molecular mechanisms of various diseases, many challenges remain in the development of minimally invasive therapies with optimal drug dosing and duration. In particular, this refers to the disorders that involve the blood vessels growth that is normally tuned by the equilibrium between pro- and anti-angiogenic factors [1,2]. Since the recognition of the fundamental role of vascular endothelial growth factors (VEGFs) in vasculogenesis and angiogenesis, many efforts have been devoted to the design of molecules that modulate VEGF-dependent signaling pathways by controlling active VEGF concentration. This therapeutic intervention is particularly important for the treatment of ischemic and inflammatory disorders, age-related macular degeneration, psoriasis, and cancer, where false regulation of VEGF signaling contributes to the progression of the disease [3,4,5]. 

Vascular endothelial growth factors are expressed as numerous splice isoforms of VEGFA, VEGFB, VEGFC, VEGFD, and placenta growth factor (PlGF). Two more VEGFs are produced by zoonotic parapoxvirus (VEGFE) and some species of snakes (VEGFF) [6]. This structural versatility affords multiple signaling outcomes even with the same VEGF receptor [7]. VEGFA is a key mediator of angiogenesis. The alternative splicing of the *VEGFA* gene is affected by hypoxia, shear stress, oncogenes, and tumor suppressor genes, thereby leading to the production of various VEGFA isoforms of different lengths, including VEGFA_111_, VEGFA_121_, VEGFA_165_, VEGFA_189_, VEGFA_206_, and VEGFA_x_ [7]. In physiological conditions, the active VEGF exists as a dimer with two receptor binding sites with glycosylated monomers bound by disulfide bonds in the antiparallel way [8]. 

**Figure 1:**
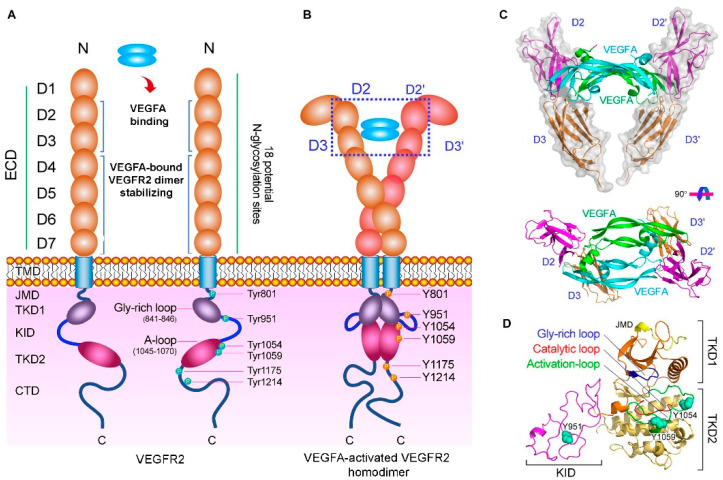
(**A**) Diagram of the VEGFR2 structure. VEGFR2 is composed of an extracellular domain (ECD) with seven Ig-like subdomains (D1-7), a transmembrane domain (TMD), a juxtamembrane domain (JMD), a catalytic tyrosine kinase domain (TKD) including ATP binding domain (TKD1), kinase insert domain (KID) and phosphotransferase domain (TKD2), and a flexible C-terminal domain (CTD). (**B**) VEGFA-activated VEGFR2 homodimer. VEGFA binding to VEGFR2 results in the phosphorylation of several tyrosine residues in TKD. (**C**) Molecular structure of VEGFA binding to D2 and D3 of VEGFR2 (PDB ID: 3V2A). (**D**) Molecular structure of TKD of VEGFR2 including TKD1 (N-lobe), KID, and TKD2 (C-lobe) (PDB ID: 4ASD) Adapted from [11], Frontiers, 2020.

Several VEGF receptors have been identified, including VEGFR1, VEGFR2, and VEGFR3. The VEGFR1 and VEGFR2 regulate physiological angiogenesis and vascular permeability, whereas the VEGFR3 drives lymphangiogenesis mediated by VEGFC/D [9]. The VEGFR2, which is expressed in vascular endothelial cells, is the main receptor for angiogenic actions of VEGFA, VEGFC, VEGFD, and VEGFE. It is a member of the tyrosine kinase superfamily and is composed of an extracellular part with seven immunoglobulin-like domains (D1-7), a single transmembrane region (TMD), a juxtamembrane domain (JMD), a split tyrosine kinase domain (TKD), and a C-terminal tail (Figure 1) [10,11].

The interaction of the receptor with VEGFs leads to the receptor dimerization and phosphorylation of specific tyrosine residues of the intracellular region followed by activation of downstream signaling pathways, which involve various signaling molecules and affect cell migration, organization, proliferation, and differentiation. In addition to VEGFRs, VEGFs bind to neuropilin co-receptors NRP1 and NRP2 and glycosaminoglycans, such as heparin, syndecans, and perlecans, thereby modulating the biological output of VEGF-mediated signaling [12,13,14].

VEGFA is the most studied growth factor of the VEGF family. Several strategies have been developed for targeting VEGFA signaling pathways for the treatment of angiogenesis-dependent diseases. These approaches include inhibition of the VEGFA secretion, neutralization of VEGFA with aptamers, antibodies, soluble VEGFRs, and the use of small-molecule inhibitors of VEGFA–VEGFR interaction or inhibitors of the tyrosine kinase activity of the receptor [15,16]. In principle, the inhibition of VEGFA–VEGFR interaction may be achieved with (i) a molecule that interacts with the receptor-binding domain of VEGFA or (ii) a molecule that binds to the recognition surface of the receptor. In this case, the former mode of inhibition is preferable, because of the risk of affecting other signaling pathways by blocking the interaction of the receptor with other natural ligands that are involved in processes other than angiogenesis [17,18,19]. In addition, extracellular VEGFA can be blocked more easily than membrane-bound receptor since there is no need to penetrate tissue to target it. Nevertheless, several VEGFR inhibitors are used in medicine, such as ramucirumab for certain advanced cancers [20].

Among clinically approved anti-VEGFA drugs, antibodies (mAbs) and soluble receptors (decoy receptors) are the most widely used, especially in ophthalmology. Bevacizumab (Avastin^®^), a full-length mAb against VEGFA, initially approved for the treatment of advanced carcinomas, has been used extensively also for age-related macular degeneration (AMD) and other chorioretinal vascular disorders [21,22]. Monoclonal antibody ranibizumab (Lucentis^®^), which binds all isoforms of VEGFA, was designed specifically to treat neovascular AMD [23,24]. High-affinity brolucizumab (Beovu^®^) is a recently approved anti-VEGFA single-chain antibody fragment for the treatment of neovascular AMD [25]. Many attempts were made to design VEGFA inhibitors based only on the binding domains of the VEGFR. In this way, several VEGF-traps were developed, including aflibercept (Eylea^®^ and Zaltrap^®^), which consists of the second Ig-like domain of VEGFR1 and the third domain VEGFR2, fused to the Fc portion of IgG1 [26,27]. Pegaptanib (Macugen^®^), a targeted anti-VEGFA aptamer developed by Eyetech Pharmaceuticals and Pfizer, was approved by FDA for patients with choroidal neovascularization. Another VEGFR-derived chimerical protein conbercept (Lumitin^®^), which is—similar to aflibercept—able to bind in addition to VEGFA also PlGF and VEGFB, was constructed by fusing VEGFR1_D2_ and VEGFR2_D3,D4_ extracellular domains with the Fc region of a human immunoglobulin IgG1 [28].

Despite all the achievements, many drawbacks of the existing VEGFA inhibitors limit their use in anti-angiogenic therapy. The systemic application of anti-angiogenic drugs can cause multiple side effects, such as vascular disorders in healthy organs or impaired wound healing. The inhibition of VEGFA signaling may also lead to the development of resistance to the therapy due to the involvement of compensatory VEGFA-independent pathways of angiogenesis. The intravitreal injections of anti-VEGFA drugs for the retinal treatment avoid systemic off-target effects, but repeated injections create a significant burden to the patients and induce rare but serious adverse effects, such as ocular infections and retinal detachment [29,30,31]. The design of novel VEGFA inhibitors and the development of the technologies to improve their pharmacological profiles remain a high-priority research area [32]. For example, the intravitreal half-life of the therapeutic antibodies and proteins was extended 3–4 fold by conjugated hyaluronan-binding peptide [33]. Another approach to avoid frequent injections of anti-VEGFA mAbs involved their delivery with sustained release systems (e.g., lipid nanoparticles) [34].

## 2. Peptide-Based Therapeutics for VEGFA Binding

In addition to the current anti-VEGF proteins, interesting therapeutic opportunities can be offered by VEGFR mimetic compounds and by glycosaminoglycan mimicking peptides, which can sequester VEGFA from the extracellular environment. The design of small peptide VEGFA inhibitors is a promising approach for the development of novel pharmaceuticals with VEGFA binding properties. Peptides are a useful starting point in the design of new pharmaceuticals for disruption of the protein–protein and protein–carbohydrate interfaces, since they can be easily synthesized and modified to meet drug-like requirements [35,36,37]. A high potential of peptide drugs resulted in the continuing growth of their number entering the clinical phase, as well as an increase in the peptide market, which is expected to reach $50B in 2025 [38].

Peptides have many advantages compared to many other small molecules. In general, they are less toxic and do not accumulate in the body due to in vivo degradation by proteases and efficient elimination. The half-life of the peptides can be easily increased by the introduction of non-coded amino acids, acylation, pegylation, cyclization, or preparation of peptidomimetics [39].

In general, antibodies and Fab-fragments have higher binding affinity to the targets compared to peptides. On the other hand, peptides can be used at higher molar concentrations than full-length antibodies and proteins, which have limitations because of the high molecular weight, solubility and aggregation issues, and low-temperature storage requirements [40,41,42]. Aggregation may lead to immunogenicity and loss of drug activity in vivo, in particular at the sites where long retention at body temperature is needed in therapy (e.g., ocular injections). From the formulation viewpoint, peptides can be more easily incorporated into controlled drug delivery systems at adequate stability and concentrations.

**Figure 2:**
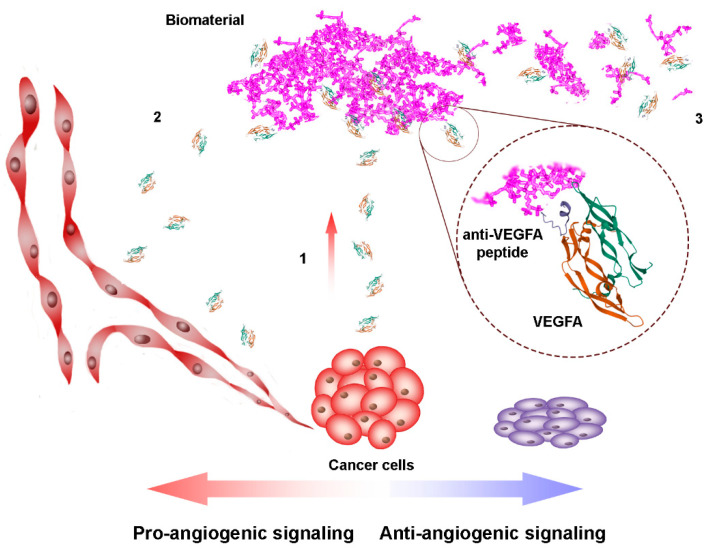
A scheme of pro- and anti-angiogenic action of VEGFA-binding biomaterials (PDB 1KAT was used for the representation of peptide/VEGFA complex). (**1**) The expression of VEGFA by cancer cells and its sequestering by the biopolymer with the anti-VEGFA peptide. (**2**) The release of VEGFA from slowly degrading biopolymer with the anti-VEGFA peptide induces pro-angiogenic signaling. (**3**) Anti-angiogenic effect and cell death are induced by the fast degrading biomaterial with the VEGFA-binding peptide.

Furthermore, encapsulation or binding of the peptides to the biopolymeric support can lead to sustained drug release and prolonged pharmacological action [43]. In this case, the biomaterial composition, its degradation rate, and the mode of peptide attachment play important roles. An interplay of various processes on the biomaterial surface can take place. Possible mechanisms of up- and downregulation of growth factor signaling by VEGFA binding biomaterials were discussed in detail by Belair et al. [44]. Allosteric VEGFA sequestering by anti-VEGFA peptide or biomaterial, where the receptor-binding domain of VEGFA is not involved, may enhance the pro-angiogenic effect since the growth factor is still able to bind and activate the receptor. On the contrary, the sequestering via the active site of the VEGFA molecule may increase or decrease angiogenic activity, depending on the affinity of the VEGFA binding ligand. When the affinity is low, sequestering biomaterial may simply enhance the residence time of the growth factor and locally enrich the cell environment with VEGFA by releasing it and accelerating by that pro-angiogenic signaling. On the contrary, the high affinity of the peptide ligand, which blocks the active site of VEGFA, may favor the anti-angiogenic effect by preventing the interaction VEGFA–VEGFR. Biomaterial degradation is also an important issue. When the peptide ligand is covalently attached to a slowly degrading biopolymeric support, the normal VEGFA inactivation by endocytosis of the VEGFA/peptide complex is prevented. Thus, instead of blocking VEGFA signaling, it may promote angiogenesis by forming a local VEGFA depot with following slow release of the growth factor (Figure 2). On the contrary, the fast degradation of the biopolymer with anti-VEGFA peptide can ensure sequestering and elimination of VEGFA from the extracellular matrix, resulting in an anti-angiogenic activity. Thus, careful fine tuning of the structures of anti-VEGFA peptide and biopolymeric support is necessary for reaching the desired drug actions.

Short peptides and peptidomimetics, as well as peptide-based biomaterials, which can bind VEGFA with high affinity, are promising alternatives to the current full-length monoclonal antibodies and soluble receptors. They can improve the existing strategies for the treatment of VEGFA-dependent diseases in terms of the effectiveness, rational use of the pharmaceuticals, and patient burden. To date, a number of peptide inhibitors of VEGFA for various biomedical applications have been described. Herein, we summarize the most interesting examples of anti-VEGFA peptides identified so far and highlight the progress and perspectives in this field of drug research.

### 2.1. Peptides as Glycosaminoglycan Mimics

Highly sulfated glycosaminoglycans (GAGs), in particular, heparin, heparan sulfate, and chondroitin sulfate derivatives play important roles in the regulation of VEGF concentration and serve as potent inhibitors of pathologic growth of blood vessels [45,46,47,48]. The binding to GAGs leads to the accumulation of the growth factors in proximity to the cell surface, thus generating a growth factor reservoir. The interaction with GAGs can also change the signaling properties of VEGFs [49]. Nevertheless, the function and influence of the single components and the structural units of these natural polymers on angiogenesis are still unclear.

**Figure 3:**
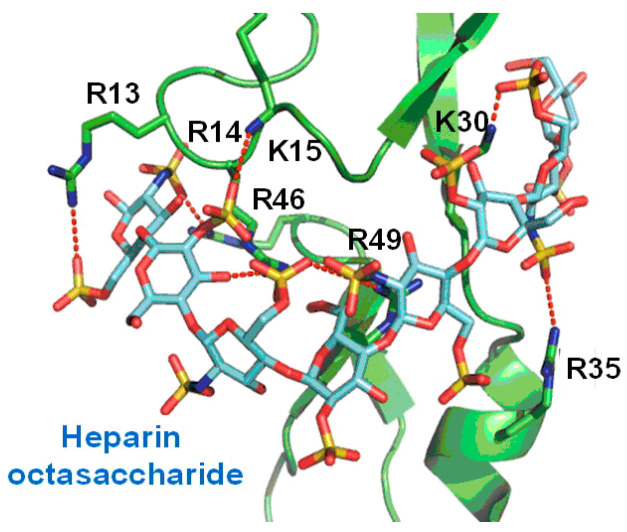
Binding model of the complex between heparin octasaccharide and heparin-binding domain of VEGFA_165_ Adapted with permission from [53], American Chemical Society, 2013.

The most abundant sulfated glycosaminoglycan, heparin, typically consists of a repeating sulfated disaccharide unit (2-O-sulfoiduronic acid and 6-O-sulfo-N-sulfo-glucosamine). The free sulfate groups of heparin are responsible for its negative charge and promote electrostatic interaction with positively charged clusters of basic amino acids (arginine and lysine) of the growth factors [50]. In addition, hydrogen bonding and hydrophobic interactions also contribute to the binding [50]. The heparin-binding domain has been identified in the C-terminal part of the VEGFA_165_ isoform of VEGFA [51]. Removal of the last 55 amino acid residues does not significantly affect the VEGFA_165_ affinity to its receptor, but leads to decreased bioactivity. The studies on modulation of angiogenesis by VEGFA binding heparin-based oligosaccharides have shown promising results in vitro and in vivo [52,53,54,55,56]. However, the inherent heterogeneity of heparin, caused by the different molecular weights and sulfation patterns of polysaccharide chains, complicates the further development of heparin-based pharmaceuticals. Therefore, the research was carried out to determine minimal structural elements of heparin, which are necessary for VEGFA binding. It was shown that the minimum size among the heparin oligosaccharides, which were able to bind VEGFA_165_, was an octasaccharide. Tetradecasaccharide was able to bind VEGFA_165_ with an affinity comparable to that of heparin (Figure 3) [57].

Due to the structural similarity, short peptides can mimic and substitute GAG molecules or their fragments in various biomedical applications. In particular, this refers to the peptides with the same functional groups as those present in GAGs, namely, sulfate, carboxyl, and hydroxyl functionalities (Tyr(SO_3_H), Asp, Glu, Ser, Thr residues). Short sulfated peptides with Tyr(SO_3_H)-X-Tyr(SO_3_H) consensus sequences, where X is an arbitrary amino acid, were shown to mimic heparin-like activity (Table 1) [58].

A combinatorial approach and analysis of about 6600 tetrapeptides allowed the identification of the best binders with this pattern. Among them, Ser-Tyr(SO_3_H)-Asp-Tyr(SO_3_H) showed 100 times stronger interaction with VEGFA_165_ than heparin mimic suramin [59]. Interestingly, the peptide with this sequence and N- and C-terminal tetraglycines retained the affinity to VEGFA_165_ without any significant anticoagulant effects. However, it was also shown that the length of the peptide and variation in the positioning and number of the sulfate groups can affect binding affinity. For example, the peptide with multiple VEGFA_165_-binding domains was less active than a peptide with a single domain. Likewise, an increasing number of sulfate groups had a negative effect on VEGFA_165_ affinity. Thus, detailed structural characteristics of the peptides are extremely important for the tight VEGFA binding, and the steric hindrance, unfavorable repulsion, or entropic factors have to be taken into consideration in the design of small peptide heparin mimetics [59].

**Figure 4:**
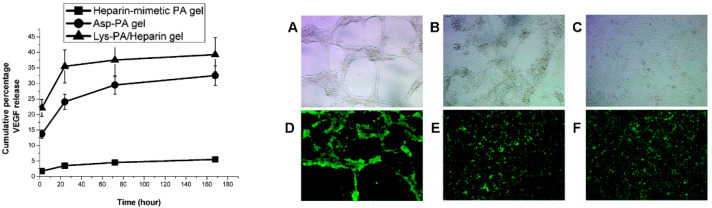
(Left) The release of VEGFA from heparin-mimetic peptide amphiphile (PA) gels. (Right) In vitro angiogenesis assay. Bright-field images of HUVECs cultured on heparin-mimetic PA nanofiber matrix (**A**), Asp-PA nanofiber matrix (**B**), and bare tissue culture plate (**C**). Cell viability assay: heparin-mimetic PA nanofiber matrix (**D**), Asp-PA nanofiber matrix (**E**), and bare tissue culture plate (**F**) Adapted with permission from [62], American Chemical Society, 2011.

However, the formation of supramolecular structures on the base of heparin-like VEGFA-binding peptides may favor angiogenesis instead of blocking it. Self-assembling heparin-like peptide amphiphiles (PAs) were shown to mimic the functions of the natural extracellular matrix to provide cell adhesion, proliferation, and differentiation [60,61]. These peptides formed a nanofibrous network and interacted with VEGFA with a binding constant similar to that of heparin [62]. The study showed a burst release of the growth factor from heparin and peptide amphiphile (PA) without the sulfonate group (Asp-PA) over two hours, whereas the release rate was significantly lower for heparin-mimetic PA (HM-PA) nanogel, which also promoted the formation of capillary-like structures of HUVECs without exogenous VEGFA (Figure 4).

This peptide scaffold was able to entrap VEGFA to form a VEGFA depot and stimulated the VEGFA signaling pathways more robustly than bare cell culture, probably, due to the autocrine signaling [62,63]. More recently, a similar glycopeptide with sulfate, carboxyl, and hydroxyl functionalities was designed to bind multiple growth factors including VEGFA_165_ [64]. In this case, highly sulfated monosaccharide was conjugated to the peptide moiety via copper(I)-catalyzed alkyne–azide cycloaddition. This glycopeptide amphiphiles self-assembled into nanoscale filaments due to the hydrophobic collapse of aliphatic tails and β-sheet formation and exhibited a concentration-dependent multivalent interaction with VEGFA_165_, which was even stronger than that of heparin. Due to the resemblance to the heparin structure, this glycopeptide could mimic GAGs and promote angiogenesis. Thus, despite a potent VEGF binding, the biological action of the heparin-like peptides depends on their supramolecular organization and they can induce pro-angiogenic response via autocrine signaling or due to the formation of VEGF reservoir, where VEGF is slowly released over time. This has to be taken into account in the design of VEGFA inhibitors on the base of heparin peptide mimics.

**Table 1:** Heparin-like VEGF-binding peptides.

Seq. ID	Structure	Activity Constant	Ref.
-	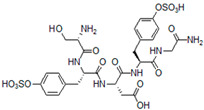	K_D_ = 3.1 ± 0.7 μM (surface plasmon resonance, SPR)	[58]
SP_a_	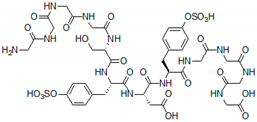	K_D_ = 0.907 ± 0.118 μM (SPR)	[59]
HM-PA	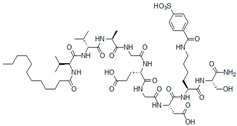	K_a_ = 2.93 ± 0.512 μM (solution form) (isothermal titration calorimetry, ITC)	[62]
PA1	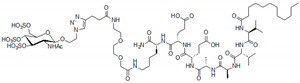	K_D_ = 0.0139 ± 0.0035 μM (SPR)	[64]

### 2.2. Peptides as VEGFR Mimics

Very interesting therapeutic opportunities may be reached by the design of the short peptides that mimic the VEGFA-binding domains of the VEGFR. It was shown that the part of the receptor that encompasses N-terminal immunoglobulin-like extracellular ligand-binding domains (ECD) can bind VEGFA as effectively as the native receptor, and the domains 2 and 3 of VEGFR2 are necessary for the binding (Figure 1C) [11,65,66]. The 101-amino acid peptide corresponding to the single domain 2 was synthesized by the solid-phase approach and it folded correctly after disulfide bridge formation. Nevertheless, the binding of the single D2 to VEGFA_165_, was 60–100 times weaker than that of the intact ECD [67].

The receptor-recognition site of VEGFA is large and topologically irregular with > 800 Å^2^ of VEGFA surface binding to a single receptor [68]. The relatively shallow surfaces that form the VEGFA–VEGFR2 interface complicate the development of small molecules which are intended to target this interaction. In the very first studies, cellulose-bound and overlapping 13-mer peptide libraries with sequences derived from the third globular domain of VEGFR2 allowed the identification of the peptides that can bind to VEGFA_165_ with high affinity. In particular, the fragment ^247^RTELNVGIDFNWEYPASK^261^ of the receptor (peptide Je-7) was found to inhibit microvascular endothelial cell (MVEC) proliferation with IC_50_ of 0.1 μM (Table 2) [69].

Along with two highly conserved residues Trp^258^ and Pro^261^, Asp^255^ was shown to be a key residue for VEGFR2 binding and, probably, to contribute to the different binding modes of various VEGF receptors. The dimerized peptide Je-11 ([RTELNVGIDFNWEYPAS]_2_K) bound VEGFA_165_ with an IC_50_ of 0.5 μM. However, it inhibited MVECs proliferation with lower efficiency than the monomeric peptide because of the occupation of only one of the binding centers of VEGFA_165_ for steric reasons. Receptor autophosphorylation, cell proliferation, and migration assays showed that both peptides had antagonistic activity only in the presence of exogenous VEGFA_165_. Further modifications of these peptides, including D-substitutions, resulted in increased serum stability and affinity to VEGFA_165_, leading to more potent inhibition of angiogenesis [70]. In particular, the best D,L-peptides blocked VEGFA_165_–VEGFR binding in the nanomolar range and inhibited the sprouting of capillary-like structures seven times better than the parent peptides. The structure of the branch strongly influenced the affinity of the dimers immobilized on the hydrogel microspheres as a carrier [71]. The insertion of two additional lysines immediately before the branching point led to the removal of about 60% of VEGFA_165_ (K_d_ = 40 pM) from the solution in serum-free conditions. However, in the presence of the serum, a release of the bound growth factor from the carrier was accelerated due to its interaction with multiple VEGFA-binding serum proteins, such as sVEGFR1, sVEGFR2, and α_2_-macroglobulin [72,73]. These peptide-based biomaterials were able to function in two different modes. They could downregulate VEGFA signaling through growth factor sequestering or upregulate it via VEGFA binding and following sustained release. This approach was successfully used for the preparation of the biomaterials, which modulated VEGFA activity dynamically over time [74,75]. The peptides were attached to PEG hydrogel microspheres with different degradation rates. Rapidly degrading microspheres reduced VEGFR2 activation in vitro and neovascularization in vivo, while microspheres with no inherent degradability functioned as a VEGFA depot by entrapping growth factor, and promoted its activity in the cell culture.

Analysis of peptide libraries allowed identification of a series of arginine-rich hexapeptides, which inhibited VEGFA_165_ binding to VEGFR with IC_50_ up to 2 μM (RRKRRR) [76]. Hexaarginine showed weaker inhibitory activity (IC_50_ = to 3.8 μM), whereas hexalysine was not active. RKKRKR had a weaker VEGFA_165_ binding (IC_50_ = to 3.4 μM) than RRKRRR, thus confirming the importance of a specific amino acid sequence, rather than just a positive charge of the peptide. In addition, it was shown that the arginine-rich peptides have the same or overlapping binding domains on the VEGFA_165_ molecule, and the interaction involves both ends of the VEGFA_121_ fragment. Interestingly, despite the positive charge, the peptides showed only limited interaction with heparin at the physiological conditions and did not prevent its interaction with VEGFA_165_. The arginine-rich peptide RRKRRR inhibited VEGFA_165_-induced neovascularization in rabbit cornea and prevented both growth and metastases of human colon carcinoma cells, demonstrating a powerful anti-angiogenic effect [74]. In combination with itraconazole, which altered the signaling pathway of VEGF stimulation, RRKRRR was used for the preparation of the nanoparticles with multivalent binding interactions with VEGFA_165_, thus enhancing their anti-angiogenic activity [77]. To enhance the proteolytic stability of the peptide, all-D-derivative (rrkrrr) was prepared [78]. In that case, the half-life of the peptide in serum was increased up to 27 times. Interestingly, the strong interaction with VEGFA_165_ and pharmacological activity were preserved in the rrkrrr peptide, even though it was not a clean retro-inverso peptide. On the contrary to RRKRRR, the dimerization of this peptide resulted in more potent inhibition of endothelial cell proliferation and migration, as well as in vivo growth of VEGF-secreting colorectal cancer cells [79]. Another approach to improving the stability and bioactivity of anti-angiogenic peptides was described by Chan et al. [80]. It was based on the design of a non-toxic cyclic peptide framework, which combined two anti-angiogenic epitopes to elicit their synergistic effect by targeting different angiogenesis pathways. Natural cyclic disulfide-rich peptides with high thermal and enzymatic stability were chosen as templates for the insertion of the anti-angiogenic peptide sequences, resulting in non-toxic and stable molecules with nanomolar potency. In particular, in combination with *β*-turn-derived peptide from somatostatin (YwKV), RRKRRR inhibited the proliferation of HUVEC and NT-29 cell lines similarly or more efficiently than anti-angiogenic drugs cilengitide and sunitinib. However, comparing to cilengitide and sunitinib was perhaps not a good choice as both are not very good inhibitors of HUVEC proliferation [81].

To find peptides that specifically bind VEGFA_165_, a random 7-mer peptide library was screened by biopanning [82]. Two peptides (WHKPFRF and WHLPFKC) were found to bind VEGFA with micromolar affinity and to inhibit HUVEC proliferation in a dose-dependent manner. Interestingly, their sequences did not present any homology with the primary sequence of VEGFR and, probably, mimicked a discontinuous VEGFA-binding site of the receptor with the amino acid residues brought to spatial proximity by receptor folding.

**Figure 5:**
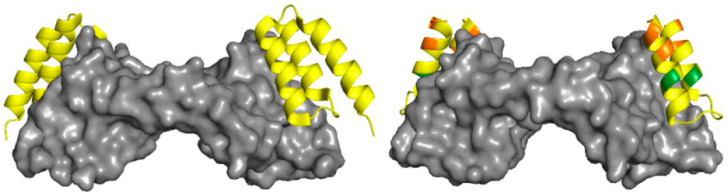
Cartoon representation of crystal structures of α-peptide Z-1-2 bound to VEGFA_8–109_ (PDB ID: 3S1K) (left) and α/β-VEGF-1 bound to VEGFA_8–109_ (right) Residues in α/β-VEGF-1 are colored by type of residue (yellow for α, green for α-aminoisobutyric acid (Aib), and orange for cyclic β). VEGFA_8–109_ is depicted as a gray surface Adapted with permission from [84], PNAS, 2015.

Furthermore, the screening of anti-VEGFA peptides by the phage display method led to the design of a series of compounds, which could bind VEGFA with high affinity and selectivity. In particular, two classes of the peptides with two- or three-helix bundles were identified after several routes of selection with the libraries against VEGFA_8-109_ [83]. The resolution of the crystal structure showed their interaction with receptor-binding regions of VEGFA_8-109_ located at the two poles of the homodimer (Figure 5) [84].

The peptides bound VEGFA_8-109_ with IC_50_ values in the nanomolar range (Table 2), but shorter peptide (mini-Z) was rapidly cleared from the bloodstream via renal elimination. In contrast, ^18^F-labeled triple-helix Z-3B showed clinically relevant prolonged retention in plasma and was successfully used as a probe to monitor VEGFA levels in the growing ovarian tumor model. Pharmacokinetic positron emission tomography images with this peptide were comparable with those obtained using anti-VEGF antibody B20. Further optimization of the pharmacokinetics of Z-domain peptides was achieved by chain shortening and iterative introduction of non-proteinogenic amino acids, in particular, β^3^-residues, cyclic β-residues, and α-aminoisobutyric acid (Aib) [68,84]. Additional stabilization of the secondary structure was ensured with the disulfide bond. Thus, a series of two-helix α/β peptides with enhanced proteolytic stability was obtained. The half-life for one of the most promising peptides α/β-VEGF-2 in the presence of proteinase K was increased up to 670 min.

**Figure 6:**
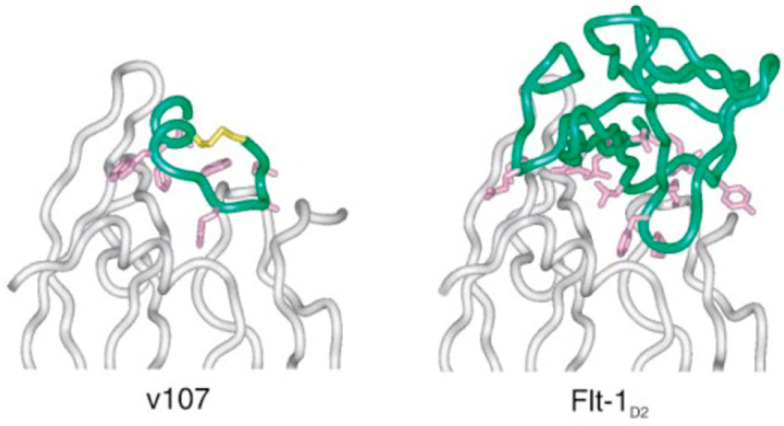
Comparison of the VEGFA_8-109_-v107 complex (**left**) and the VEGFA_8-109_-Flt-1_D2_ (VEGFA_8-109_-VEGFR-1_D2_) complex (**right**). The VEGFA_8-109_ ribbon is colored grey, while the v107 and Flt-1_D2_ (VEGFR-1_D2_) ribbons are colored green with the side-chains in contact with VEGFA shown in magenta Adapted with permission from [86], Elsevier, 2002.

Another series of VEGFA-binding peptides identified by the phage display method was described by Fairbrother et al. [85]. In particular, IC_50_ values measured by SPR for two most promising peptides v107 (GGNEc[CDIARMWEWEC]FERL) and its analog v114 (VEPNc[CDIHVMWEWEC]FERL) were 0.70 and 0.22 μM, respectively. The structure of the complex VEGFA_8-109_-v104 was studied in detail by NMR [86]. It was shown that the peptide has a mixed turn-helix conformation with hydrophobic residues faced toward the VEGFA_8-109_ molecule, and it consists of a disordered N-terminus (residues 1–4), a type-I turn at residues 6–9, an extended region from residues 9–12, and a C-terminal α-helix from residues 13–19. It interacts with the receptor-binding region of VEGFA, thus competing with the receptor for VEGFA binding (Figure 6).

Later, the analysis of peptide libraries showed that W(E/D)W(E/D) is a consensus core motif and the peptides with this amino acid sequence can be considered as mimotopes that mimic VEGFA binding regions of VEGFR [87,88]. The study of a series of short cyclic D-peptides confirmed this finding and only c(D-Pro-Trp-Glu-D-Pro-Trp-Glu) bound VEGF_11-109_ in the low-millimolar range [89]. The high potential of v107 and v114 as VEGFA_165_-binding peptides was also proven in the design of the bioanalytical tools for VEGFA_165_ detection and the screening of other compounds that associate with the receptor-binding surface of the VEGFA dimer [90,91].

To improve the affinity and enzymatic stability of the peptide v114, several modifications were carried out. In particular, v114* (in which the methionine residue was replaced by a norleucine residue) was able to bind VEGFA_165_ with a K_i_ of 60 nM. Alanine scans of v107 and v114 and a “β-scan” of v114* were used to determine the positions of the amino acids that are crucial for VEGFA_165_ binding and, thereafter, a series of peptidomimetics on the base of v114* was prepared and investigated [92]. However, the introduction of various modifications and peptide shortening did not increase VEGFA affinity and inhibition properties, although the authors concluded that the reduction in conformational flexibility or the introduction of fluorinated ^16^Phe could improve VEGFA binding properties [93].

The conformational constraint was achieved by the introduction into v114* of non-coded α-tetrasubstituted amino acid Aib, which is known to stabilize the folded peptide structures [94]. The insertion of Aib into the N-terminal tail or in position 12 of the amino acid sequence improved the inhibition of VEGFA_165_-induced proliferation of HUVEC cells from IC_50_ = 20 μM (v114*) up to IC_50_ = 4 μM. In addition, Aib modification increased the enzymatic stability of the peptide. Interestingly, VN peptide with shortened N-tail showed a stronger VEGFA_165_ binding than v114* and the peptide without N-terminal residues. This may indicate the importance of the N-terminal tail for the stabilization of the conformation necessary for VEGFA_165_-binding, despite the absence of direct interaction with the VEGFA_165_ molecule.

**Figure 7:**
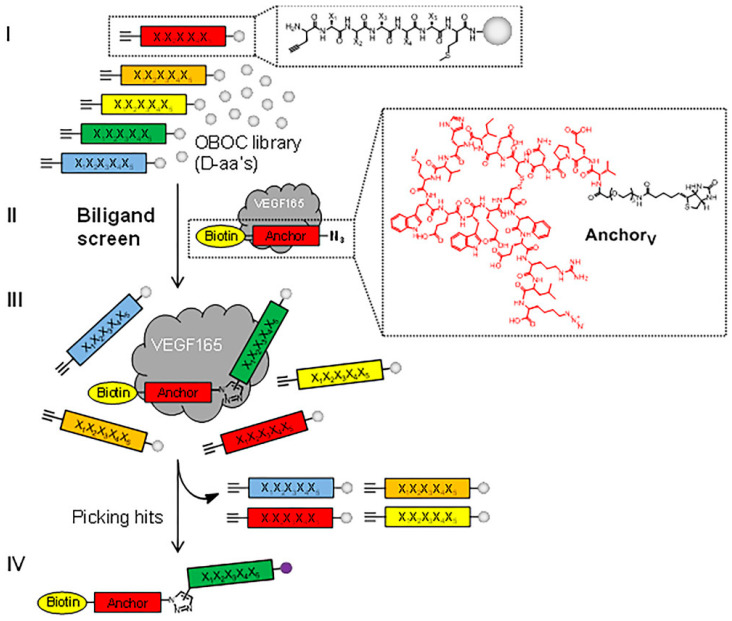
In situ click screening of VEGFA_165_. A randomized pentapeptide library (X = D-amino acid) is synthesized on TentaGel resin. D-propargylglycine is fixed at the N-terminus, and D-methionine is fixed at the C-terminus (**I**). This library is incubated with VEGFA_165_ and biotin-labeled AnchorV in a biligand screen (**II**). Target binding is detected by an anti-VEGFA_165_ antibody followed by alkaline phosphatase (AP)-conjugated secondary antibody. The hit beads are washed, stripped, and reprobed with AP-conjugated streptavidin to detect products of the target-catalyzed in situ click chemistry (**III**,**IV**). Methionine-specific CNBr cleavage and sequencing by MALDI-TOF/TOF yield the sequences of biligand candidates. The biligand candidates are synthesized on a larger scale and assayed to assess in vitro performance (affinity, selectivity, stability, etc.). Repeating the process once yields triligands and twice results in tetraligands Adapted from [95], John Wiley & Sons, 2016.

Another improvement of the binding affinity of v114* to VEGFA_165_ was achieved by using the protein catalyzed capture agent method (Figure 7) [95].

**Table 2:** Peptides as VEGFR mimics.

Seq. ID	Sequence	VEGF Binding	Ref.
Je-7	RTELNVGIDFNWEYPASK	IC_50_ = 0.1 μM (proliferation assay)	[69]
Je-11	(RTELNVGIDFNWEYPAS)_2_K	IC_50_ = 0.5 μM (proliferation assay)	
-	EfaylIDFNWEYPASK	IC_50_ = 1 μM (competition assay)	[70,71,72,73,74,75]
-	(EfaylIDFNWEYPAS)_2_K	IC_50_ = 0.03 μM (competition assay)	
-	RRKRRR	IC_50_ = 10 μM (inhibition assay)	[76,77,78,79,80]
-	rrkrrr	IC_50_ = 10 μM (inhibition assay)	
MCoAA-01	CGRKRRRGCRRDSDCGACICRYwKVCGSGSDGGV	IC_50_ = 10.83 ± 0.09 μM (inhibition assay, HUVECs)IC_50_ = 40.12 ± 0.13 μM (inhibition assay, NT-29)	
WHK7	WHKPFRF	IC_50_ ~ 0.5 mM (proliferation assay)	[82]
WHL7	WHLPFKC	IC_50_ ~ 0.5 mM (proliferation assay)	
Mini-Z-1	FNKECLLRYKEAALDPNLNLYQRIAKIVSIDDDC	IC_50_ = 227 nM (ELISA)	[83]
Z-1-2	VDNKFNKEMHNAYAIEIALLPNLNDQQFHAFIWSLIDDPSQSANLLAEAKKLNDAQAPK	IC_50_ = 343 nM (ELISA)	
Z-3B	VDNKFNKEMQNAYAIEIALLPNLNGSQTFAFITSLRDDPSQSANLLAEAKKLNDAQAPK	K_D_ = 55 nM (Bio-Layer Interferometry)	
α/β-VEGF-1	VDNKFNKEXc[CNZRAIEUALDPNLNDQQFHUKIWZIIXDC]	K_i_ = 0.11 μM (competitive fluorescence polarization assay)	[68,84]
α/β-VEGF-2	VDNKFNKEXc[CNZRAIEUALDPNLNDUQFHUKIWZIIXDC]	K_i_ = 0.39 μM (competitive fluorescence polarization assay)	
21	KFNKEXc[CNZRAIEUALDPNLNDUQFHUKIWZIIXDC] where D, F = β^3^-residues, X, Z = cyclic β-residues and U = Aib	K_i_ = 0.15 μM (competitive fluorescence polarization assay)	
v107	GGNEc[CDIARMWEWEC]FERL	IC_50_ = 0.70 ± 0.06 μM (SPR)	[85]
v114	VEPNc[CDIHVMWEWEC]FERL	IC_50_ = 0.22 μM (SPR)	
v114*	VEPNc[CDIHV^n^LWEWEC]FERL	K_i_ = 0.06 μM (competitive fluorescence polarization assay)	[92]
-	VXPXc[CDIHV^n^LWXWEC]FZRX	K_i_ = 1.6 μM (competitive fluorescence polarization assay)	
-	XEXNc[CDIHV^n^LXEWXC]FZRX, where X = β^3^-residues, Z = cyclic β-residue	K_i_ = 4.6 μM (competitive fluorescence polarization assay)	
Aib2	VAibPNc[CDIHV^n^LWEWEC]FERL	EC_50_ = 10.0 ± 1.1 μM (proliferation assay)	[94]
Aib12	VEPNc[CDIHV^n^LWAibWEC]FERL	EC_50_ = 3.5 ± 0.7 μM (proliferation assay)	
kv114*	KAibKKc[CDIHV^n^LWEWEC]FERL	EC_50_ = 6.0 ± 0.4 μM (proliferation assay)	
VN	VNc[CDIHV^n^LWEWEC]FERL	EC_50_ = 4.0 ± 0.5 μM (proliferation assay)	
Bi-L_v_	X-VEPNCDIHVMWEWECFERL-Tz4-lfrew	-	[95]
Tri-L_v_	X-VEPNCDIHVMWEWECFERL-Tz4-lfrew-Tz4-eeird	EC_50_ = 2.6 ± 0.5 nM (ELISA)	
Tetra-L_v_	X-VEPNCDIHVMWEWECFERL-Tz4-lfrew-Tz4-eeird-Tz4-qfkyr where X = biotin-PEG3 label Az4 = L-azidolysine	EC_50_ = 0.74 ± 0.05 nM (ELISA)	

In situ click chemistry on the VEGFA_165_ molecule of azide-modified v114* and a D-peptide from the peptide library led to the formation of a biligand with a higher affinity to VEGFA_165_ than that of the v114* and D-peptide alone. The iterative addition of the third and fourth D-peptide to v114* yielded a further increase in VEGFA_165_ inhibition, which was comparable to that of bevacizumab in the case of the tetraligand. Along with high affinity, the peptides showed improved stability against proteases and slow clearance from the peritoneal cavity after intraperitoneal injection. However, they were rapidly removed from the bloodstream after intravenous administration.

## 3. Conclusions and Perspectives

The research carried out during recent decades has allowed peptides to be selected that can interact with vascular endothelial growth factor A and to inhibit its angiogenic activity with high efficiency. In this review, we summarized the most interesting examples of VEGFA-binding peptides. These peptides can be divided into two groups, namely, heparin mimics and the peptides disrupting the interaction of VEGFA with its receptor. The optimization of their structures resulted in potent peptide inhibitors and peptidomimetics with a high affinity to VEGFA. The properties of these peptides were further improved to ensure high enzymatic stability and to prolong drug retention in biological fluids. Encapsulation or binding of the peptides to the biopolymeric support was shown to be a promising approach to prolong the pharmacological action on VEGFA. Depending on the design, either anti-angiogenic or pro-angiogenic efficacy may be tailored to such systems. A careful design of the biomaterial and optimization of the mode of attachment of the peptide ligand can ensure the desired anti-angiogenic outcome. Thus, peptide inhibitors of VEGFA hold a great promise as alternatives to the currently used pharmaceuticals in many medical indications.

## Data Availability

Not applicable.
